# Generation of a ceramide synthase 6 mouse lacking the DDRSDIE C-terminal motif

**DOI:** 10.1371/journal.pone.0271675

**Published:** 2022-07-18

**Authors:** Jiyoon Kim, Yael Pewzner-Jung, Tammar Joseph, Shifra Ben-Dor, Anthony H. Futerman

**Affiliations:** 1 Department Biomolecular Sciences, Weizmann Institute of Science, Rehovot, Israel; 2 Life Sciences Core Facilities, Weizmann Institute of Science, Rehovot, Israel; Universite Paris Diderot-Paris7 - Batiment des Grands Moulins, FRANCE

## Abstract

The important membrane lipid, ceramide, is generated by a family of homologous enzymes, the ceramide synthases (CerSs), multi-spanning membrane proteins located in the endoplasmic reticulum. Six CerS isoforms exist in mammals with each using a subset of acyl-CoAs for (dihydro)ceramide synthesis. A number of mice have been generated in which one or other CerS has been genetically manipulated, including complete knock-outs, with each displaying phenotypes concomitant with the expression levels of the CerS in question and the presumed biological function of the ceramide species that it generates. We recently described a short C-terminal motif in the CerS which is involved in CerS dimer formation; deleting this motif had no effect on the ability of the CerS to synthesize ceramide *in vitro*. In the current study, we generated a CerS6 mouse using CRISPR-Cas9, in which the DDRSDIE motif was replaced by ADAAAIA. While levels of CerS6^ADAAAIA^ expression were unaffected in the CerS6^ADAAAIA^ mouse, and CerS6^ADAAAIA^ was able to generate C16-ceramide *in vitro*, ceramide levels were significantly reduced in the CerS6^ADAAAIA^ mouse, suggesting that replacing this motif affects an as-yet unknown mechanism of regulation of ceramide synthesis via the DDRSDIE motif *in vivo*. Crossing CerS6^ADAAAIA^ mice with CerS5 null mice led to generation of viable mice in which C16-ceramide levels were reduced by up to 90%, suggesting that depletion of C16-ceramide levels is compensated for by other ceramide species with different acyl chain lengths.

## Introduction

Ceramide is a critical lipid, not only since it is found at the hub of the sphingolipid (SL) biosynthetic pathway [1], but also because it is involved in a number of intracellular signaling pathways [2]. Ceramide is generated *de novo* via the sequential action of four enzymes, namely serine palmitoyl transferase, 3-ketosphinganine reductase, (dihydro)ceramide synthase (CerS) and dihydroceramide reductase [3]. All four of these enzymes are located in the endoplasmic reticulum [4] where they are subject to various modes of regulation [3].

The CerS were initially identified ~20 years ago, first in yeast [5] and subsequently in mammals [6]. The distinguishing feature that defines each of the six members of the CerS family in mammals is their *N*-acyl CoA specificity, with each CerS using a defined subset of acyl CoAs [7]. For instance, CerS2 uses very-long chain acyl CoAs such as C22:0-C24:1 [8] whereas CerS5 and CerS6 both use long chain CoAs such as C14:0–16:0 [9, 10]. While the CerS are highly specific for the acyl CoAs that each use, they are more promiscuous with respect to the sphingoid long chain base; thus, CerS can *N*-acylate both sphinganine generated from the *de novo* synthesis pathway to generate dihydroceramide (d18:0), along with the sphingosine that is produced in the degradative (recycling) pathway to generate ceramide (d18:1) [9, 11].

The CerS are part of a larger gene family, the Tram-Lag-CLN8 (TLC) domain family [12, 13]. We recently identified a short DxRSDxE motif at the C terminus of CerS [14] which is not found in other members of the TLC family, and is the best way of defining the CerS phylogenetically. Deletion of this motif had no effect on the ability of CerS to generate (dihydro)ceramide in *in vitro* CerS assays, or via metabolic labeling using NBD-sphinganine (NBD-Sph), but did affect the ability of CerS to form homo- or heterodimers as determined by co-immunoprecipitation, leading to the suggestion that the DxRSDxE motif is involved in CerS dimer formation [14].

To further explore the physiological role of the DxRSDxE motif, we have now generated, using CRISPR-Cas9, a mouse in which this motif in CerS6 (DDRSDIE) has been replaced by ADAAAIA. The CerS6^ADAAAIA^ mouse is able to synthesize ceramide *in vitro* at levels similar to the CerS6 wild type (WT) mouse. However, C16:0-ceramide and C16:0-dihydroceramide levels, measured by mass spectrometry, were 30–40% lower in the small intestine (jejunum) and in the liver compared to the CerS6 mouse, which is similar to the extent of C16-ceramide reduction in the CerS6 null mouse [15]. Moreover, when the CerS6^ADAAAIA^ mouse was crossed with CerS5 null mice, C16:0-ceramide and C16:0-dihydroceramide levels were depleted by up to 90%, although the mice survived into adulthood. The molecular basis by which ceramide levels are depleted in the CerS6^ADAAAIA^ mouse is unknown, but limits the usefulness of this mouse to determine the precise role that the DxRSDxE motif plays in CerS *in vivo*. However, the viability of the CerS6^ADAAAIA^/CerS5 null mice, despite the ~90% depletion of C16-ceramide levels, suggests it could be a useful tool to delineate the precise roles of C16-ceramide *in vivo*.

## Materials and methods

### Reagents

NBD-sphinganine and palmitoyl-CoA (C16-CoA) were from Avanti Polar Lipids (Alabaster, AL). Defatted-bovine serum albumin, a protease inhibitor cocktail and an anti-CerS6 antibody were from Sigma-Aldrich (St. Louis, MO). Horseradish peroxidase was from the Jackson Laboratory (Bar Harbor, ME). An enhanced chemiluminescence (ECL) detection system was from Cyanagen (Bologna, Italy). An anti-glyceraldehyde 3-phosphate dehydrogenase (GAPDH) antibody and silica gel 60 thin layer chromatography plates were from Merck (Billerica, MA). All solvents were of analytical grade and purchased from Bio-Labs (Jerusalem, Israel).

### Mice

C57BL/6JOlaHsd mice were purchased from Envigo (Ness Ziona, Israel) and used to generate CerS6 ^ADAAAIA^ mice by CRISPR-Cas9 [16]. The CRISPR guide sequence was 5′- AGCTAGATTCAATGTCACTC-3′. The guide sequence was chosen by optimizing the lowest off-target [17, 18] and maximum on-target potential [18–20]. The single strand repair oligo was 5’-ACCCTGCTGGTCCTAGGAAGCTGGTGACACTCCCATCTTTGTATTCTGCAGGTATCCAAGGCTGACGCGGCTGCCATTGCATCTAGCTCAGATGATGAGGACTCAGAGCCTCCAGGGAAGAAACCACACTCTTCAACAACC-3’. Primers used for genomic screening were as follows: forward 5′-CATTATGTTCCTGCACCACC-3′; reverse 5′-ATTCACAGACAGGACACATG-3′, to yield a 702 base pair fragment that was cleaved by either BsaWI or BsrDI restriction endonucleases to identify the WT allele or edited alleles, respectively. Mouse sequences were analyzed using Sequencher v5.4 (GeneCodes Corp. Ann Arbor, MI, USA) and visualized using the University of California, Santa Cruz Genome Browser [21].

CerS5^tm2b(KOMP)Mbp^ (CerS5 null) mice (stock #022819) were purchased from Jackson Laboratories (Bar Harbor, ME, USA). Primers for genotyping were as follows: CerS5, forward 5’-GATGGGAGTTTTTCTTTGTGC-3’; reverse, 5’- TGATGGATTGGGCCTAGTGT-3’; CerS5 null forward 5’- CGGTCGCTACCATTACCAGT-3’, reverse 5’-TTGCCTCAAAACCCACCTAC-3’. CerS6 ^ADAAAIA^ mice were mated with CerS5 null mice to generate CerS6 ^ADAAAIA^/CerS5 null mice.

### Ethics statement

Animals experiments were approved by the Weizmann Institute of Science Animal Care Committee. Mice were maintained under special pathogen-free conditions and treated according to the Animal Care Guidelines of the Weizmann Institute of Science Animal Care Committee and the National Institutes of Health’s Guidelines for Animal Care.

### CerS assays

CerS assays were performed as described [22, 23]. Mouse tissues were homogenized in 20 mM Hepes-KOH, pH 7.2, 25 mM KCl, 250 mM sucrose and 2 mM MgCl_2_ containing a protease inhibitor cocktail (Sigma-Aldrich). Protein was determined using the BCA reagent (Cyanagen). Cell homogenates were incubated with 15 μM NBD-Sph, 20 μM defatted-bovine serum albumin and 50 μM C16-CoA in a 20 μl reaction volume at 37°C. Reactions were terminated by addition of chloroform/methanol (1:2, v/v) and lipids extracted [24]. Lipids were dried under N_2_ and resuspended in chloroform/methanol (9:1, v/v) and applied to thin layer chromatography plates (20x10 cm Silica Gel plates, Merck). The thin layer chromatography plates were developed using chloroform/methanol/2M NH_4_OH (40:10:1, v/v/v). NBD-labelled lipids were visualized using an Amersham Typhoon 5 biomolecular fluorescence imager and quantified by ImageQuantTL (GE Healthcare, Chalfont St Giles, UK).

### Lipidomics

Tissues were homogenized in double-distilled water containing protease inhibitors using a GentleMACS Dissociator (Miltenyi Biotec, Bergisch Gladbach, Germany). Ceramide and dihydroceramide levels were determined by liquid chromatography electrospray ionization tandem mass spectrometry (LC-ESI-MS/MS) using an ABI 4000 quadrupole-linear ion trap mass spectrometer [14, 25]. Groups were composed of 3 females and 1 male, except for the CerS6 ^ADAAAIA^ group, which used 2 females and 2 males.

### Western blotting

Western blotting was performed as described [14]. Mouse tissues were lysed in 1% Nonidet P40 containing lysis buffer (137 mM NaCl, 1 mM EDTA, 5% glycerol in 20 mM Tris-HCl, pH 7.2) supplemented with a protease inhibitor cocktail. Proteins were separated by SDS-PAGE and transferred to nitrocellulose membranes. CerS6 was identified using a rabbit anti-CerS6 antibody (1:1,000 dilution) and goat anti-rabbit horseradish peroxidase (1:10,000 dilution) as the secondary antibody. Equal loading was confirmed using a mouse anti-GAPDH antibody (1:5,000 dilution) followed by incubation with a goat anti-mouse horseradish peroxidase antibody (1:10,000 dilution). Proteins were detected using the ECL detection system and quantified using the ChemiDoc MP imaging system (Bio-Rad, Hercules, CA).

## Results and discussion

**Fig 1:**
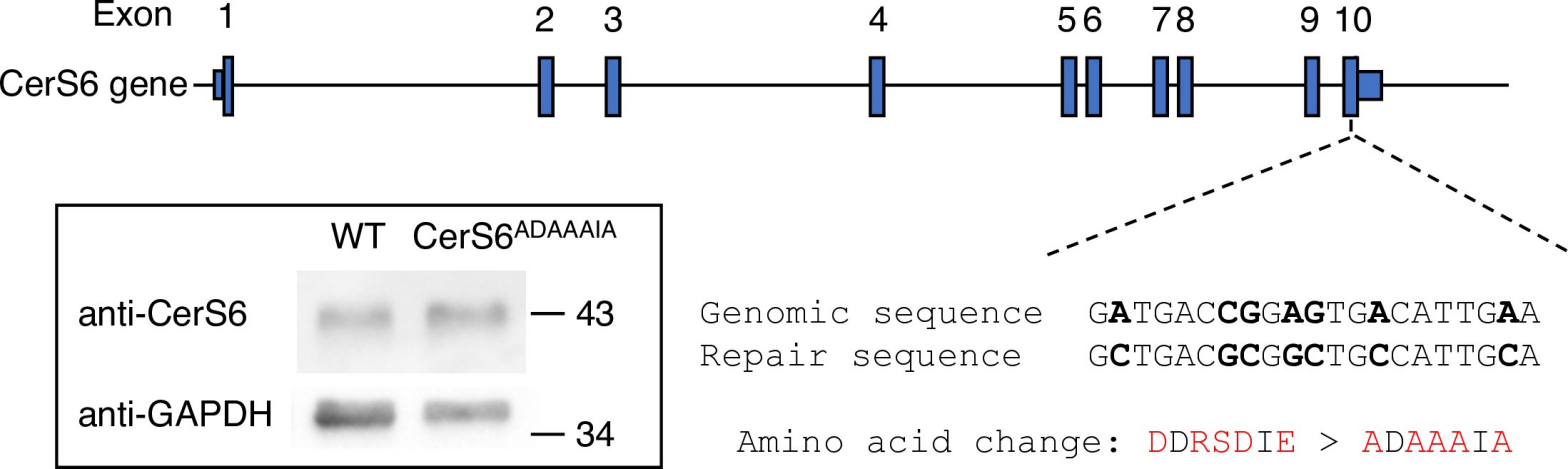
Generation of CerS6^ADAAAIA^ mice. The CerS6 genomic region showing the CRISPR/Cas9 design for editing the CerS6 DDRSDIE motif. Genomic location (mm10 chr2:68,861,441–69,114,282) of the guide RNA cleavage site is shown as a dashed line. The single strand (ssDNA) repair oligo spanning 60 base pairs of the genomic sequence up- and down-stream of the edited motif is indicated. The genomic and repair sequences are shown, and amino acid residues that were modified are in *red*. *Insert*, mouse kidney homogenates (20 μg of protein) were analyzed by Western blotting using an anti-CerS6 antibody, repeated twice with similar results. Mr markers are shown. GAPDH was used as a loading control.

CerS6^ADAAAIA^ mice were generated using CRISPR-Cas9 (Fig 1). CRISPR/Cas9 edited mice were mated with WT C57BL/6 mice to generate heterozygous mice which were then crossed to generate homozygous CerS6^ADAAAIA^ mice, which were born with normal Mendelian distribution and no apparent phenotype. Levels of CerS6^ADAAAIA^ expression, examined by Western blotting, were similar to levels of CerS6 in the kidney (Fig 1) (where CerS6 is expressed at high levels [15]).

**Fig 2:**
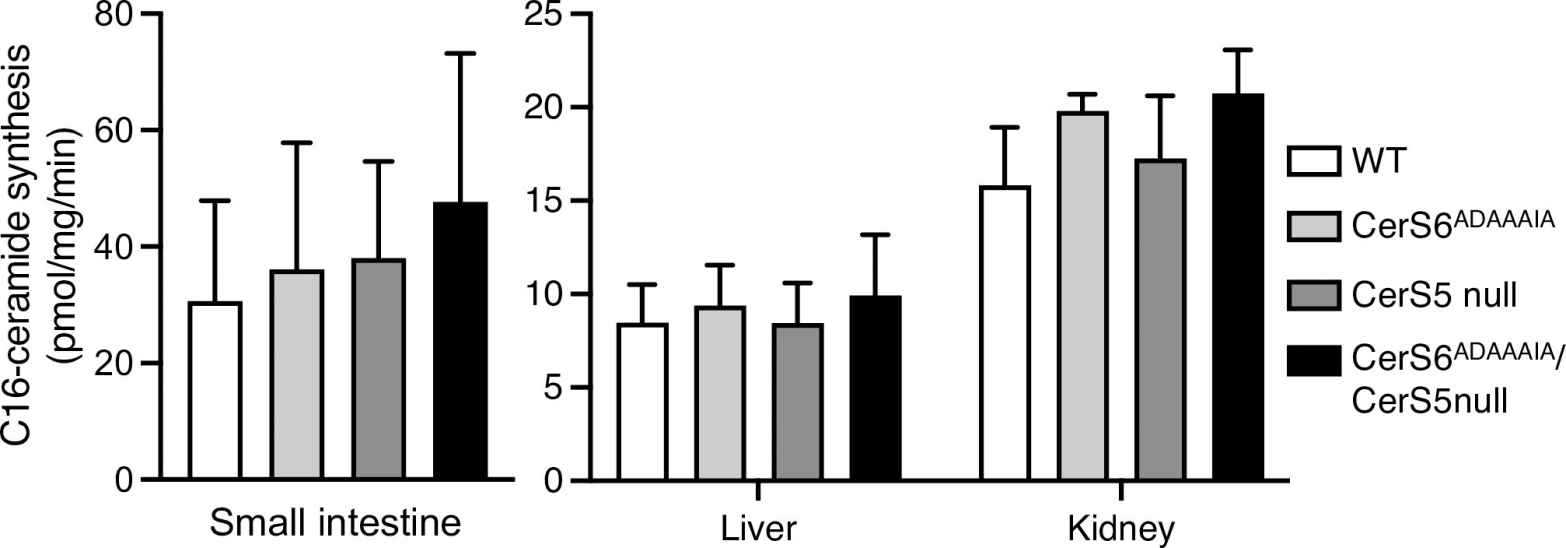
*In vitro* ceramide synthase activity in tissues from the CerS6^ADAAAIA^ mouse. CerS activity was assayed using 20 μg of protein from homogenates from the indicated tissues, and C16:0-CoA was used as substrate. Data are means ± S.D., n = 4. No significant differences were detected.

We next analyzed the activity of CerS6^ADAAAIA^ *in vitro* in homogenates obtained from a number of tissues from the CerS6^ADAAAIA^ mouse. No difference in levels of C16-ceramide synthesis were observed between CerS6 and CerS6^ADAAAIA^ mice (Fig 2). However, since C16-ceramide can also be generated by CerS5 [26], and since both can be expressed in the same tissues, we also determined levels of ceramide synthesis in tissues from a CerS5 null mouse, and in tissues obtained upon crossing CerS6^ADAAAIA^ mice with CerS5 null mice. No differences in levels of ceramide synthesis were detected in homogenates from any of these mice (Fig 2). In the case of the CerS6^ADAAAIA^ mouse, this data confirms previous observations [14] that deletion of this motif does not affect ceramide synthesis *in vitro*. When only CerS5 is knocked-out, presumably CerS6 continues to generate C16:0-ceramide, and likewise in tissues from CerS6^ADAAAIA^/CerS5 null mice, we assume that C16:0-ceramide is generated via the action of CerS6^ADAAAIA^ *in vitro*.

**Fig 3:**
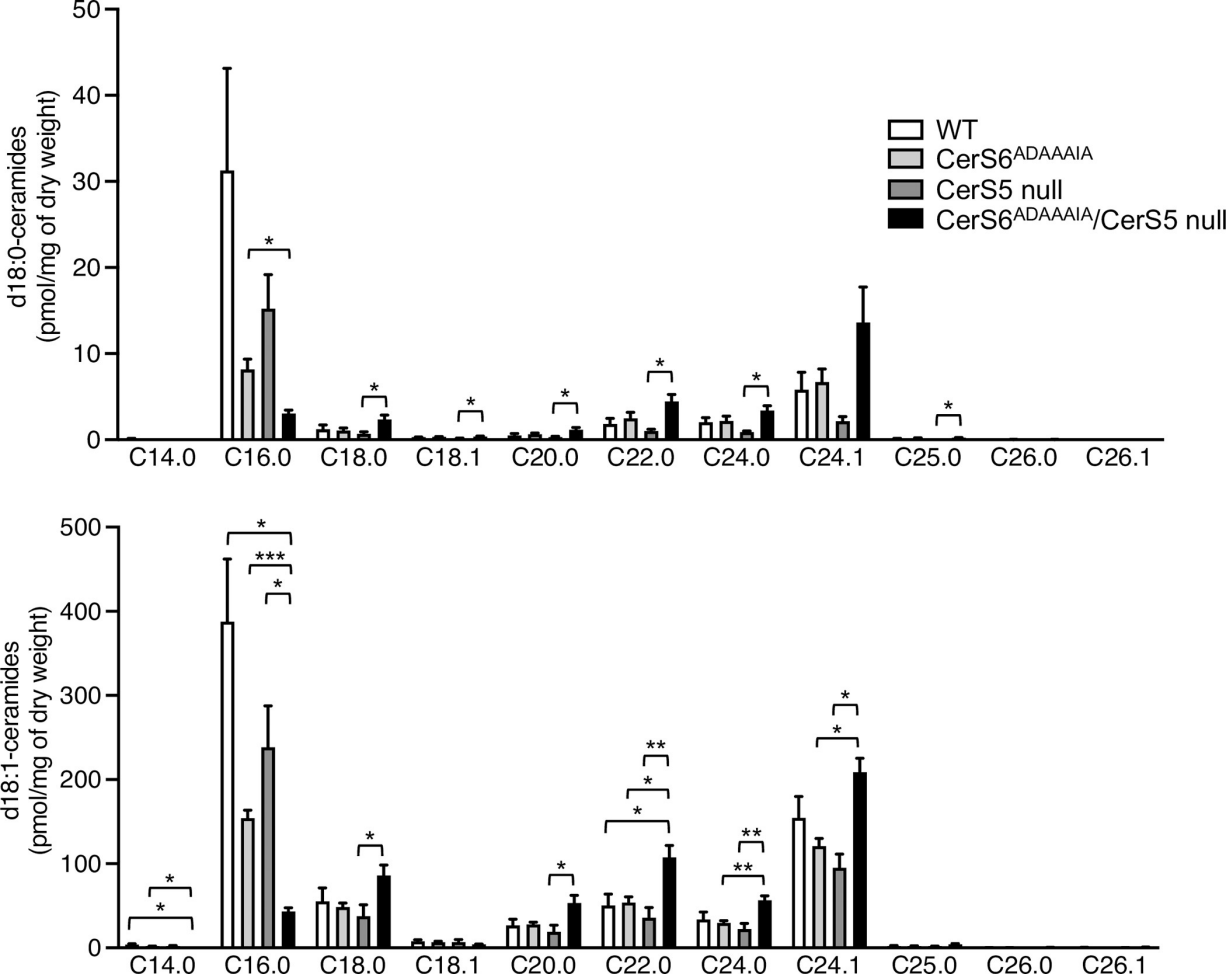
Dihydroceramide and ceramide levels in the small intestine of the CerS6^ADAAAIA^ mouse. Fatty acyl composition of d18:0- and d18:1-ceramides were measured by LC-MS/MS. Data are means ± S.E.M., n = 4. *, p<0.05; **, p<0.01; ***, p<0.001.

**Fig 4:**
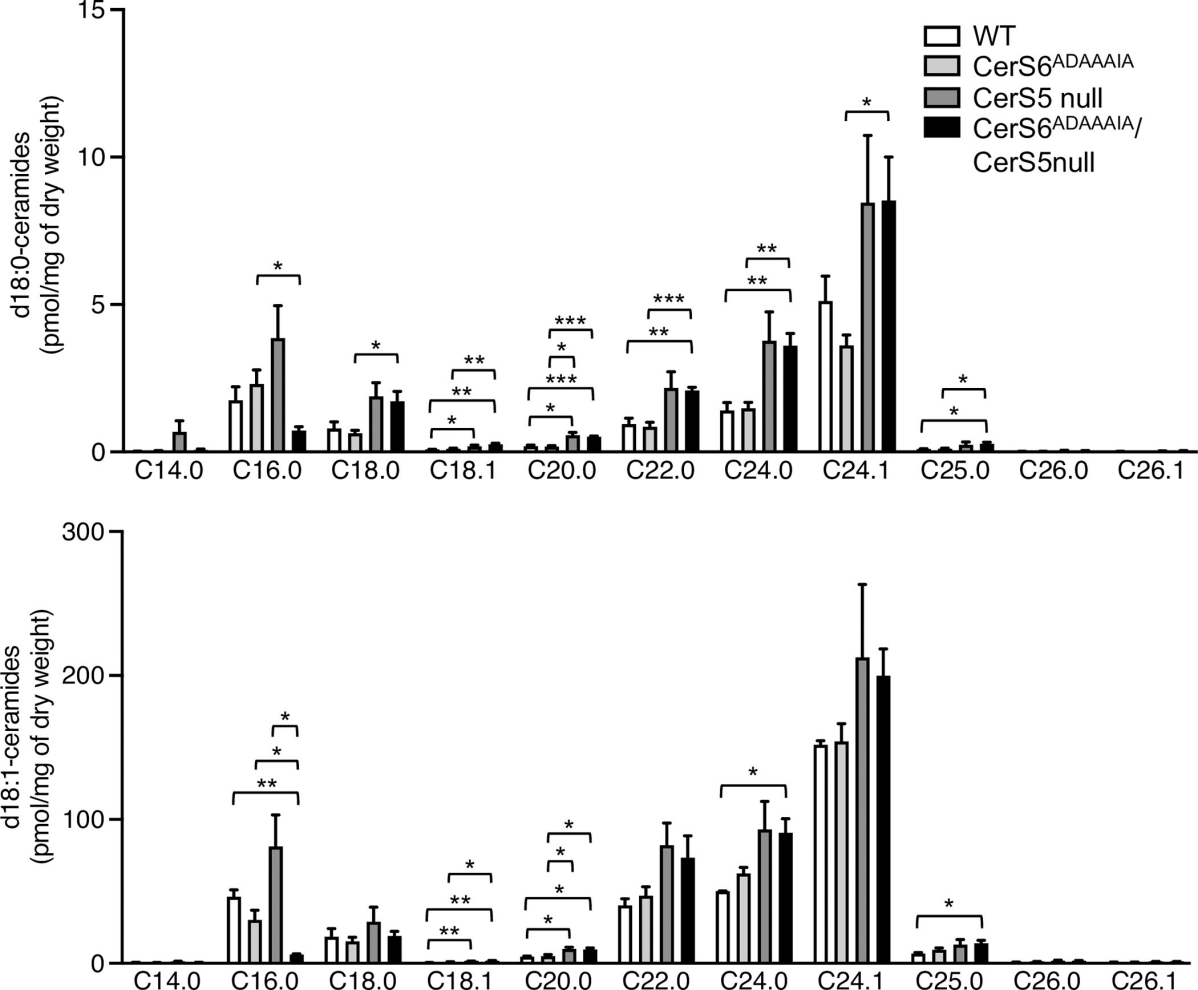
Dihydroceramide and ceramide levels in the liver of CerS6^ADAAAIA^ mouse. Fatty acyl composition of d18:0- and d18:1-ceramides were measured by LC-MS/MS. Data are means ± S.E.M., n = 4. *, p<0.05; **, p<0.01; ***, p<0.001.

Since CerS6^ADAAAIA^ is able to generate ceramide *in vitro*, we presumed that there would be few if any changes in the ceramide composition of tissues from the CerS6^ADAAAIA^ mouse, although unexpectedly this was not the case. Thus, both C16:0-dihydroceramide and C16:0-ceramide levels were decreased in the small intestine (jejunum) (Fig 3), by ~60% in CerS6^ADAAAIA^ mice, although no clear-cut reduction was detected in liver (Fig 4), similar to that observed in the CerS6 null mouse [15]. As expected, C16:0-dihydroceramide and C16:0-ceramide levels were decreased in the small intestine from CerS5 null mice, but not in the liver, presumably due to different expression levels of CerS5 and CerS6 in each tissue [7], and a much larger reduction than in CerS6^ADAAAIA^ mice was seen in the small intestine from CerS6^ADAAAIA^/CerS5 null mice compared to CerS5 null mice, with C16:0-ceramide and C16:0-dihydroceramide levels close to zero (Fig 3), with a similar decrease in liver (Fig 4). In CerS6^ADAAAIA^/CerS5 null mice, very-long chain ceramide and dihydroceramide levels were elevated, as is often seen in CerS null mice where reduction in levels of a ceramide species with one particular *N*-acyl chain length frequently leads to elevation of ceramides with a different *N*-acyl chain length [27]. While this latter observation is expected, the reduction of ceramide and dihydroceramide levels upon replacing the DDRSDIE motif of CerS6 with ADAAAIA is quite unexpected based on the ability of CerS6^ADAAAIA^ to synthesize ceramide *in vitro*, and thereby limits the usefulness of this mouse in studies to delineate the role of the DDRSDIE motif *in vivo*. However, the CerS6^ADAAAIA^/CerS5 null mouse could act as an excellent resource to determine the roles of C16-ceramide *in vivo*, due to the reduction of C16-ceramide levels by ~90%.

A number of mice have been generated in which CerS have been genetically modified, although most of them were generated so as to completely delete the CerS in question [15, 28–30]. Only one study is available which documents attempts to generate a mouse in which a CerS2 functional domain, namely the Hox-like domain [31] (amino acid residues 79 to 120), was deleted [32]. Similar to our study, and prior to attempting to generate a mouse lacking the Hox-like domain, CerS2^Δ79–120^ was confirmed to be catalytically active *in vitro*, although with a small reduction in activity [32]. Unlike the current study, CerS2^Δ79–120^ was not expressed due to exon skipping in exons 3 and 4 [32]. Similarly, we generated CerS2^Δ84–118^ mice, which also resulted in exon skipping, and lack of protein production (unpublished studies). We also generated CerS2 mice with the C-terminal DXRSD motif replaced by alanine residues, as well as mice lacking the C-terminus, but due to intron retention, CerS2 was not expressed.

Despite the inability in previous studies to generate CerS mice defective in one or other functional domain, we nevertheless felt that the DDRSDIE motif in CerS was an attractive option to attempt to test a functional role of a particular domain *in vivo*. Supporting this notion was the observation that the CerS6^ADAAAIA^ mouse was able to generate C16-ceramide *in vitro*, although, unfortunately it was unable to generate ceramide *in vivo*. This is somewhat reminiscent of previous observations that levels of ceramide in cultured cells upon deletion, or manipulation of various functional sequences in CerS does not always correspond to direct enzymatic assays *in vitro* [33, 34]. This suggests that additional, currently-unknown mechanisms of CerS regulation exist, which may depend on a number of factors. CerS interact with a number of proteins *in vitro* and *in vivo* [35, 36], and moreover are regulated by a number of post-translational mechanisms, including phosphorylation [33, 34]. It may be that these mechanisms are not required for CerS activity *in vitro*, but that their *in vivo* function affects CerS activity in such a way that cannot be reproduced *in vitro*. For example, FATP2 which interacts with CerS2 in mouse liver, increases ceramide levels upon overexpression in Hek293T cells [36], and a number of proteins involved in regulation of acyl CoA, such as ACSL5 [37] and ELOVL1 also interact with the CerS *in vivo* [38]. Together, our findings suggest that novel approaches will be required to determine the role of functional domains of CerS *in vivo*, at least in mouse models, which is likely to lead to further refinement of our understanding of their complex modes of regulation.

## Supporting information

S1 Raw images Images of original western blot used in Fig 1. (PDF)

## Abbreviations

SLs: sphingolipids
CerS: ceramide synthase
GAPDH: glyceraldehyde 3-phosphate dehydrogenase
NBD: Sph, NBD-sphinganine
WT: wild type
