# Supplementary figures and images for: Generation of a ceramide synthase 6 mouse lacking the DDRSDIE C-terminal motif
Source: PLoS One. 2022 Jul 18;17(7):e0271675. doi: 10.1371/journal.pone.0271675 (PMC9292091; doi:10.1371/journal.pone.0271675)

CerS6

WT CerS6<sup>ADAAAIA</sup>

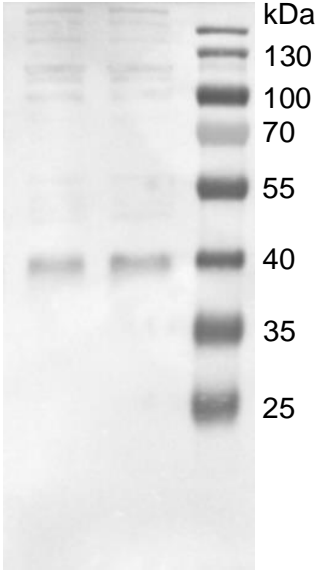

GAPDH

WT CerS6<sup>ADAAAIA</sup>

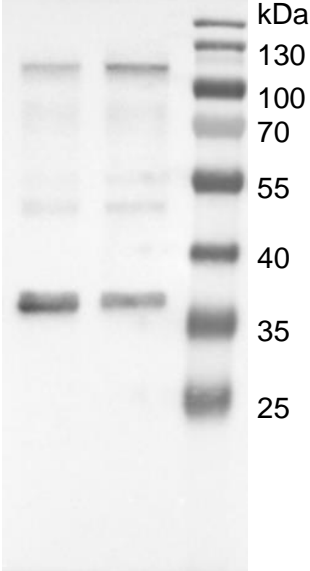

Supplement: S1 Raw images — (PDF) [file pone.0271675.s001.pdf]
